# Electoral campaign contributions: an obstacle to sugary drink industry regulation in Brazil?

**DOI:** 10.1017/S1368980021005036

**Published:** 2022-11

**Authors:** Aline Brandão Mariath, Larissa Galastri Baraldi, Ana Paula Bortoletto Martins

**Affiliations:** 1 Chamber of Deputies, Brasília, Distrito Federal, Brazil; 2 Post-graduate Program in Public Health Nutrition, School of Public Health, University of São Paulo, São Paulo, Brazil; 3 Center for Food Studies and Research, University of Campinas, Campinas, Brazil; 4 Center for Epidemiological Studies in Health and Nutrition, University of São Paulo, São Paulo, Brazil

**Keywords:** Corporate political activity, Food policy, Legislation, Public policy, Sugar-sweetened beverages

## Abstract

**Objective:**

To assess corporate electoral campaign contributions from industries related to sugary drinks production and the characteristics of the elected officials financed by the sector.

**Design:**

Cross-sectional analysis of electoral campaign contributions from corporations related to sugary drinks production (sugary drink industries and sugary drink input industries) to candidates to the Chamber of Deputies, Brazil.

**Setting:**

Elections to the 55^th^ Congress (2015–2019), held in October 2014.

**Participants:**

Candidates to the Chamber of Deputies, Brazil.

**Results:**

Forty-nine companies or corporate groups that produce sugary drinks and fifty-two corporations that produce inputs for sugary drinks manufacturing contributed to electoral campaigns of candidates in the 2014 Election. Contributions from this industry sector represented 7·3 % of all corporate contributions and helped finance 11·7 % of the candidates and 46·2 % of the elected officials. The transnationals Ambev and Coca-Cola were the first and second biggest donors, respectively. Revenues mediated by political parties, from sugary drink industries and from corporate members of some industry associations (Abir, Unica and CitrusBR), were more prevalent. Among elected officials, a significant association was found between being financed by the sector and representing the south-east region, having higher education level and referring themselves as being professional politicians. In the multivariate model, financed candidates were 27 % more likely to be elected.

**Conclusions:**

Corporations related to sugary drinks production have contributed to the electoral campaigns of almost half of the Federal Deputies in Brazil in 2014. This possibly facilitates access to decision-makers and could help buy influence on legislative proposals, including health-related food policies.

There is now a wealth of evidence on the negative impact of sugary drinks on human health. Sugary drink intake has been associated with increased risk of dental caries and tooth decay, weight gain and higher body adiposity, type 2 diabetes, high blood pressure and other health problems^(1–5)^. As a result, it is widely accepted that efforts to tackle obesity and diet-related non-communicable diseases must include government regulation of sugary drink industry activities and practices, particularly because industry’s voluntary self-regulation initiatives have been shown insufficient to achieve this goal^(6–10)^. Frequently recommended public policies include sugary drinks taxation, restrictions to marketing targeted at children, adoption of warning front-of-package nutrition labels in industrialised products, among others^(11–15)^.

However, government regulation expectedly faces strong opposition from the private sector as it might impact production practices, sales and profit^(7,16,17)^. In order to prevent or postpone regulation, sugary drink industries use a considerable variety of corporate political activity (CPA) strategies and practices. Studies have shown that this industry sector is highly engaged in CPA. Practices such as sponsoring health-related organisations, funding and influencing health-related scientific research, disseminating messages in the media to shift the blame away from the sugary drink industry in the obesity epidemic, casting doubt on scientific evidence linking sugary drinks to obesity and other diet-related non-communicable diseases, using corporate social responsibility actions, lobbying against government regulation and threatening to use legal measures against government regulation have been reported^(18–24)^. CPA strategies and practices of the ultra-processed food and drink industry can be undertaken individually by companies, but when it comes to publicly opposing regulation, the literature has provided evidence that collective action through industry associations is often preferred^(17,25–27)^, as this could help mitigate reputational risks for individual companies.

Despite the recent increase in the research field addressing these issues, especially following the publication of the framework developed by Mialon *et al.*^(16)^ to identify and monitor the CPA of the ultra-processed food industry, very little is known with respect to electoral campaign contributions, which is a practice included in the ‘Financial incentive’ strategy. This strategy refers to the provision of any gifts, funds or financial incentives to politicians, political parties and other policymakers.

Corporate electoral campaign contributions can be pragmatic, with the aim of pursuing financial returns, or ideological, as a means of influencing election results. Both types of motivation can have political and economic consequences because campaign contributions are acknowledged to facilitate access to elected officials and can be used to influence political decisions in policy-making processes. In fact, campaign contributions are regarded as part of lobbying strategies because they increase the chances of lobbying success^(28,29)^.

In this paper, we aim to assess corporate electoral campaign contributions from industries related to sugary drinks production to the candidates to the 55^th^ Congress (February 2015 to February 2019) of the Chamber of Deputies, Brazil, as well as the characteristics of the elected officials financed by the sector. This research represents a unique opportunity to look into this corporate political practice in detail in the country because corporate campaign contributions – which in the 2014 election cycle represented 75 % of total electoral funds^(30–32)^ – have been forbidden since September 2015^(33)^. In addition to that, contributions from food and drink companies were the second highest prevalent in this election, running behind only the contributions from construction companies^(34)^.

## Methods

Brazil is a democratic multi-party presidential Republic which adopts a bicameral system for lawmaking process at the national level. The National Congress is comprised of the Chamber of Deputies (the lower House) and the Federal Senate (the upper House). Every 4 years, 513 representatives (Federal Deputies) are elected across each of the twenty-six States and the Federal District for a 4-year mandate in the Chamber of Deputies. Each State and the Federal District elect between eight and seventy-two representatives – the number of seats varies according to the local population. The number of candidates also varies across States and the Federal District^(35)^.

The law that regulates the elections in Brazil requires political parties and candidates to report all campaign revenues and payments. With respect to the revenues of candidates and political parties, until September 2015 corporations could contribute up to 2 % of their gross revenues in the year before the election cycle. These corporate contributions were added to government funds to political parties, personal financial resources from the candidates and contributions from citizens^(36)^. Corporations could channel their contributions directly to candidates (herein called ‘direct contributions’) or to political parties, which then would distribute the financial resources to their candidates, according to unknown criteria (herein called ‘indirect contributions’, as they were mediated by the political parties). In addition, candidates could channel their own financial resources, from any source (corporations, political party or citizens) to other candidates. This type of contribution was also called ‘indirect contribution’ in this analysis. Because most of the indirect contributions were mediated by the political parties, we opted not to distinguish the intermediary source (if the political party or other candidate).

Data on campaign contributions are publicly available from the Superior Electoral Court website^(37)^, and the dataset from the 2014 Election (held in October 2014) was downloaded in February 2020. Variables related to the candidates (name, *CPF* – ‘Individual Taxpayer Registration number’ – represented State, political party, date of birth, sex, ethnicity, marital status, schooling, occupation and electoral result) and the revenues (amount, name of the donor, registration number of the donor at the Brazilian Internal Revenue Service, and economic sector of the donor) were used. When campaign contributions were indirect (intermediated by political parties or other candidates), variables related to the original donor (name of the original donor, registration number of the original donor at the Brazilian Internal Revenue Service and economic sector of the original donor) were also collected. Contributions were converted from *Reais* (the Brazilian currency) to US$ using the mean exchange rate calculated from the election period (between July and October 2014).

After an initial exploratory analysis, the variables related to the candidates were organised as follows: (a) States and the Federal District were grouped into the regions north, north-east, center-west, south-east and south; (b) age (calculated based on the election day) was categorised as <35 years old, ≥35 and <50 years old, ≥50 and <65 years old, and ≥ 65 years old; (c) ethnicity was grouped into Caucasians and non-Caucasians (*pardos*, blacks, Asians and indigenous); (d) as for marital status, married and widowed were group, as well as separated and divorced candidates; (e) schooling was classified dichotomously according to the completion of higher education level; (f) occupation was classified dichotomously, and those who referred already being an elected official were regarded as professional politicians; (g) political parties were classified according to their coalitions as opposing the Federal Executive branch, supporting the Federal Executive branch or no coalition; and (h) the result of the election was classified dichotomously as elected and non-elected (this category included those who figured in the list of substitutes and although not having been elected could gain a seat in the Chamber of Deputies sometime during the 55^th^ Congress).

As for the variables related to revenues, they were used to identify the two groups of corporations of interest for this study: (1) *sugary drink industries*, which comprised manufacturers that had at least one type of sugar-sweetened beverage in their product portfolio (i.e. sodas, energy drinks, sports drinks, 50 % per cent fruit juices, soya-based drinks, powdered drink mixes, syrups, ready-to-drink teas, yogurts and other dairy drinks, chocolate milk powder, powdered coffee). Distribution companies that belonged to major sugary drink manufacturers were also included in this group. Corporations that only produced artificially sweetened beverages or non-sugar-added 100 % fruit juice were not included; and (2) *sugary drink input industries,* which comprised manufacturers that produce the main inputs for sugary drinks (sugars and fruit concentrates). Although food additives are largely used for manufacturing sugary drinks, companies that produce this type of input were not included in this study because of the inability to determine precisely which additives are used specifically for producing sugary drinks.

In Brazil, medium and large corporations often hold several registry numbers at the Brazilian Internal Revenue Service, especially if they operate in more than one State. This is the case, for instance, of corporations that produce sugary drinks such as Coca-Cola and Ambev. Each of their factories and distributors holds a unique registry number. Another example is that of larger sugarcane industries that own several sugarcane farms – each farm is considered a unique company and therefore holds a unique registry number, regardless of being located in the same city or not. As a consequence, corporations such as these could use more than one registry number to make their campaign contributions. In fact, this used to be a common practice. Widely distributing campaign contributions among several of their registry numbers was a strategy used, especially by large corporations to divert attention from the high amounts of money they contributed to electoral campaigns^(34)^. In addition, each company record (represented by its registry number) at the Brazilian Internal Revenue Service describes their economic activities. However, because medium and large corporations can have multiple registry numbers, economic activities listed for a single registry number at the Brazilian Internal Revenue Service might not reflect all those activities the corporation is involved in. For instance, a corporation that produces sugarcane sugar could have contributed using a registry number whose main activity was ‘sugarcane crop production’; an ultra-processed food industry which has sugary drinks among its portfolio of products could have contributed using a registry number whose main activity was ‘biscuit production’.

Taking all this into account, so as to reduce the chances of not including companies or corporations related to sugary drinks and sugary drink inputs production, we decided to adopt a broader approach when identifying corporations of interest for this analysis. The dataset from the Superior Electoral Court provides a variable that identifies the economic sector of the donor, which is based on the codes and descriptions of the National Classification of Economic Activities (NCEA) and represents the main economic activity related to its registry number at the Brazilian Internal Revenue Service (only one code from the NCEA). Firstly, we selected a wide variety of codes related to the economic sector that could potentially include a corporation of interest, regardless of whether or not they were strictly related to sugary drinks or their inputs (i.e. ‘fruit juice production’, ‘ethanol production’, etc.).

Secondly, the corporations classified under the selected codes were individually checked on the Brazilian Internal Revenue Service website^(38)^. For those that were active at the time of this research, we based inclusion on their main and secondary economic activities available at the Brazilian Internal Revenue Service website (and not on the activity reported to the Superior Electoral Court). Decision-making was also supplemented by information available at the websites of the corporations, whenever they were available. In the case of inactive corporations, classification was based preferably on the economic activity informed at the Brazilian Internal Revenue Service website. When this piece of information was not available, economic activity reported to the Superior Electoral Court was used. Registry numbers that belonged to the same corporation were clustered.

Next, corporations were classified according to the type of sugary drink or input they produced, identified from their websites. Categories of sugary drink industries were as follows: (1) dairy drinks; (2) non-dairy drinks; and (3) dairy and non-dairy drinks. These industries were also classified in non-exclusive categories related to the subtype of sugary drinks they produced, as some corporate groups have more than one type of sugary drinks in their product portfolio. Sugary drink input industries were categorised as (1) sugars or (2) fruit concentrates.

Finally, corporations were classified according to their participation in industry associations, as they often have seats in public hearings in the Brazilian Legislature and also have a history of participation and interference in public policy processes related to food and nutrition^(25,26,39)^. The associations were identified from a registry of private sector and civil society representatives kept by de 1^st^ Secretariat of the Chamber of Deputies, requested by FOI. These representatives have a facilitated access to Federal Deputies and are often invited to take part in public hearings. Selected trade associations were as follows: the Brazilian Association of Soft Drinks and Non-Alcoholic Beverages Industry (Abir), which represents Big Soda and major soft drink industries in Brazil; *Associação dos Fabricantes de Refrigerantes do Brasil (Afrebras)* and *Sindicato Nacional da Indústria de Refrigerantes (Sindirefri)*, both representing small- and medium-sized soda industries; the Brazilian Association of Citrus Exporters (CitrusBR), which represents producers and exporters of citrus juices; and the Brazilian Sugarcane Industry Association (Unica), which represents the main sugar, ethanol and bioelectricity producers in the south central of the country. Additionally, we decided to include *Viva Lácteos* – which represents dairy manufacturers and is not in the registry kept by the 1^st^ Secretariat of the Chamber of Deputies, but has recently participated in a voluntary agreement to reduce sugar content of industrialised foods^(40,41)^. Associated companies and corporate groups were identified from the industry associations’ websites.

The relevance of contributions from associated corporations was assessed as follows. First, contributions from corporations belonging to each of the selected industry associations were added up. Secondly, for each of the selected industry associations, we selected all non-affiliated corporations whose activities were strictly related to their scope and, therefore, could be potential affiliates. Next, for each association, we calculated the proportion of contributions from affiliated corporations in relation to the sum of all contributions from corporations whose activities were strictly related to their scope.

The association of categorical independent variables was analysed with chi-square test or one-tailed Fisher’s exact test, depending on their frequencies. In Brazil, both campaign revenues and payments are highly heterogeneous. The cost per vote varies a lot across States and can be influenced by the political capital of the candidate, the number of running candidates and seats in a given State, geographic and demographic characteristics of the region where a candidate’s base voters are registered, as well as the fact that elections to the Chamber of Deputies are held under a proportional representation system. As a result, in order to assess whether contributions from sugary drink and/or sugary drink input industries increased the chances of being elected, we decided not to use face values of contributions, but rather a dichotomous variable to describe whether candidates had been financed by this industry sector or not. As there was a large number of individuals who did not receive any contribution from this industry sector, a generalised linear model analysis using negative binomial regression was performed. In this analysis, only candidates who had reported at least one corporate campaign contribution were included, because being financed by the private corporate sector *per se* already increases the chances of being elected. Being financed by sugary drink and/or sugary drink input industries (yes or no) was the outcome, and the result of the election (elected or non-elected) was the exposure. The multivariate model was adjusted for sex, ethnicity, represented region, higher education level, being a professional politician, political party coalition and total campaign revenues. Data organisation and analyses were carried out using Stata IC 15^(42)^, and differences were considered statistically significant at the level of *P* < 0·05.

## Results

Forty-nine corporations that produce sugary drinks and fifty-two corporations that produce inputs for sugary drinks manufacturing contributed to electoral campaigns of candidates to the Chamber of Deputies in the 2014 Election. Among corporations that produce sugary drinks, 46·9 % only produce non-dairy drinks (*n* 23), 42·9 % produce only dairy drinks (*n* 21) and 10·2 % produce both types of drinks (*n* 5). With respect to the subtypes of sugary drinks, ready-to-drink dairy drinks are produced by 38·8 % of the corporations (*n* 19); fruit or fruit-flavoured drinks, by 24·5 % (*n* 12); sodas, by 22·4 % (*n* 11); energy drinks, by 20·4 % (*n* 10); fruit-flavoured powdered drinks and powdered dairy drinks, by 14·3 % (*n* 7); ready-to-drink teas and sports drinks, by 6·1 % (*n* 3); and fruit or fruit-flavoured concentrates, by 4·1 % (*n* 2). As for corporations that produce inputs for sugary drinks manufacturing, 96·2 % only produce sugars (*n* 50), and 3·8 % produce fruit concentrates (*n* 2) (data not shown).

Contributions from sugary drink industries and sugary drink inputs industries totalled US$ 25,131,856·92 (7·3 % of all corporate contributions) and helped finance the electoral campaigns of 11·7 % of the candidates (585 out of 4985 candidates). The distribution of the financial resources is shown in Table 1. Among sugary drink industries, contributions from non-dairy drinks industries were the most prevalent (59·9 % of the candidates), while among sugary drink inputs industries, the most prevalent contributions were those from corporations which produced sugars (85·6 % of the candidates). With the exception of contributions from corporations related to sugar production, indirect contributions to the candidates were the most prevalent.

**Table 1:** Absolute (US$) and relative (%) electoral campaign contributions from corporations that produce sugary drinks and their inputs, as reported by the candidates to the 55^th^ Congress of the Chamber of Deputies. Brazil, 2014

Industry sector	Corporations (*n*)	Candidates (*n*)	Direct contributions (US$)	Indirect contributions (US$)	Total contributions (US$)	%
Sugary drink industries						
Dairy drinks	21	44	817 232·44	1 275 833·53	2 093 065·97	12·5 %
Non-dairy drinks	23	320	1 006 536·38	9 022 004·75	10 028 541·13	59·9 %
Dairy and non-dairy drinks	5	83	912 280·69	3 709 097·63	4 621 378·32	27·6 %
Total:	49	399	2 736 049·51	14 006 935·91	16 742 985·42	100 %
Sugary drink input industries						
Sugars	50	184	5 461 723·00	1 721 872·39	7 183 595·39	85·6 %
Fruit concentrates	2	129	405 988·56	799 287·55	1 205 276·11	14·4 %
Total:	52	289	5 867 711·56	2 521 159·94	8 388 871·50	100 %
Total:	101	585	8 603 761·07	16 528 095·85	25 131 856·92	

Note: Some candidates have been financed by more than one corporation related to the industry sectors hereby analysed.

Therefore, the sum of candidates in total values does not necessarily represent the sum of the subgroups.

The top five biggest donors among sugary drink industries were as follows: (1) Ambev (US$ 6 010 392·98); (2) Coca-Cola (US$ 4 583 871·93); (3) Cervejaria Petrópolis (US$ 3 007 856·14); (4) Vigor Alimentos (US$ 877 175·43); and (5) Brasil Kirin (US$ 548 042·10). Among sugary drink inputs industries, the top five donors were as follows: (1) Atvos (US$ 1 725 913·04); (2) Coopersucar (US$ 1 534 950·00); (3) Tereos (US$ 844 250·00); (4) Cutrale (US$ 839 064·69); and (5) Grupo São Martinho (US$ 403 923·46).

Contributions from associated corporations are described in Table 2. Indirect contributions from corporations associated with *Viva Lácteos*, Abir and CitrusBr were more prevalent than those from non-associated groups. As for corporations associated with Unica, direct contributions were the most prevalent. No indirect contributions from companies associated with *Afrebras* and *Sindirefri* were identified. Contributions from corporations associated with Abir, Unica and CitrusBR were always more prevalent in comparison to non-associated ones, representing 60·6 %, 73·3 % and 69·6 % of the total contributions from corporations whose activities were strictly related to the scope of the associations. Contributions from companies associated with Afrebras and Sindirefri represented only 1·0 % and 0·1 %, respectively, of all contributions from corporations that produce soft drinks.

**Table 2:** Absolute (US$) electoral campaign contributions from associated corporations that produce sugary drinks and their inputs and their participation in relation to non-associated corporate groups, as reported by the candidates to the 55^th^ Congress of the Chamber of Deputies. Brazil, 2014

Industry association	Corporations (*n*)	Candidates (*n*)	Direct contributions (US$)	Indirect contributions (US$)	Total contributions (US$)	Participation in relation to non-associated corporations
Sugary drink industries						
Viva Lácteos	8	17	528 508·75	1 349 339·65	1 877 848·40	14·9 %
Abir	13*	308	903 036·38	9 235 179·50	10 138 215·88	60·6 %
Sindirefri	1	3	14 254·39	0	14 254·39	0·1 %
Afrebras	3	6	146 438·59	0	146 438·59	1·0 %
Sugary drink input industries						
Unica	11	137	3 629 360·75	1 517 766·85	5 147 127·60	73·3 %
CitrusBR	1	29	404 824·56	434 240·17	839 064·73	69·6 %

* Eight out of the thirteen corporations were part of the Coca-Cola System in Brazil.

As for the profile of candidates and elected Federal Deputies financed by corporations related to sugary drinks and sugary drink input industries, these are shown in Table 3. The percentages of candidates who were financed were higher among men, Caucasians, in the age group ≥ 65 years, who were married/widowed or separated/divorced, who represented the north-east region of the country, who had higher education level, who referred being professional politicians and whose political party coalition was aligned with the Federal Executive branch. Regarding the elected Federal Deputies, 46·2 % were financed by this industry sector (*n* 237), and a significant association was found for those who represented the south-east region, who had higher education level and who referred being professional politicians.

**Table 3:** Distribution of candidates and elected Federal Deputies to the 55^th^ Congress of the Chamber of Deputies according to reported electoral campaign financing from sugary drink and sugary drink input industries. Brazil, 2014

Variables	Candidates					Elected Federal deputies				
	Financed %	*n*	Non-financed %	*n*	*P*	Financed %	*n*	Non-financed %	*n*	*P*
Sex										
Males	13·6	489	86·4	3110	<0·001	92·4	219	88·0	243	0·10
Females	6·9	96	93·1	1290		7·6	18	12·0	33	
Ethnicity										
Caucasians	14·0	427	86,0	2616	<0·001	83·1	197	77·5	214	0·11
Non-Caucasians	8·1	158	91·9	1784		16·9	40	22·5	62	
Age										
<35 years old	9·9	69	90·1	625	<0·001	10·1	24	10·9	30	0·54
≥35 and <50 years old	10·0	206	90·0	1853		32·5	77	29·3	81	
≥50 and <65 years old	13·1	244	86·9	1618		45·2	107	50·4	139	
≥ 65 years old	17·8	66	82·2	304		12·2	29	9·4	26	
Marital status										
Single	7·7	105	92·3	1264	<0·001	13·9	33	15·6	43	0·86
Married or widowed	13·5	400	86·5	2554		73·8	175	72·8	201	
Separated or divorced	12·1	80	87·9	582		12·3	29	11·6	32	
Region										
North	6·6	36	93·4	509	<0·001	5·5	13	18·8	52	< 0·001
North-east	15·8	158	84·2	843		30·8	73	28·3	78	
Center-west	12·4	46	87·6	325		8·4	20	7·6	21	
South-east	11·0	269	89·0	2185		40·5	96	30·1	83	
South	12·4	76	87·6	538		14·8	35	15·2	42	
Higher education										
No	7·3	166	92·7	2094	<0·001	16·0	38	23·6	65	< 0·05
Yes	15·4	419	84·6	2306		84·0	199	76·4	211	
Professional politician										
No	8·7	380	91·3	4003	<0·001	40·5	96	56·5	156	< 0·001
Yes	34·1	205	65·9	397		59·5	141	43·5	120	
Party coalition										
No coalition	4,5	49	95·5	1033	<0·001	3·8	9	7·2	20	0·23
Situation	17·6	292	82·4	1365		59·9	142	58·7	162	
Opposition	10·9	244	89·1	2002		36·3	86	34·1	94	

The regression analysis comprised a subset of 2803 candidates who reported having received at least one corporate contribution. The results showed that being financed by corporations related to sugary drinks and/or sugary drink input industries increased the chances of being elected. In the multivariate model (adjusted for sex, ethnicity, represented region, higher education level, being a professional politician, political party coalition and total campaign revenues), those who had been financed by this industry sector were 27 % more likely to be elected (prevalence ratio: 1·27; *P* = 0·008; (95 % CI 1·06, 1·52)) than those who had not been supported (data not shown).

## Discussion

To the best of our knowledge, this study represents the first systematic attempt worldwide to address electoral campaign contributions from corporations related to the sugary drink industry tracking both donors and benefited candidates. Therefore, it fills an important knowledge gap with respect to this CPA practice. Undoubtedly, research like this can only be carried out due to the transparency of accountability reports in Brazil. Although this study only points to the tip of the iceberg with regard to corporate strategies to influence public policies in the Brazilian Legislature, it provides important insights for the understanding of this practice. In addition, transnational corporations have been identified among electoral campaign donors, and it is likely that this CPA practice could also be found internationally, especially in countries where corporate campaign contributions are allowed.

Some of the corporations identified in this study are among the biggest corporate donors of all electoral campaigns at the national level in Brazil from 2002 to 2014: Construtora Queiroz Galvão, a conglomerate that includes Queiroz Galvão Alimentos (a fruit concentrate producer), in the fifth position; Construtora Norberto Odebrecht S.A., a conglomerate that holds sugarcane sugar producers, in the ninth position; Cervejaria Petrópolis, which produces beers and soft drinks, in the eleventh position; Sucocítrico Cutrale Ltda., which produces fruit concentrates, in the fifteenth position; and Recofarma Indústrias do Amazonas Ltda., which is part of the Coca-Cola System in Brazil, in the twentieth position^(34)^.

The findings that a modest number of candidates were financed by this industry sector despite the presence of corporate groups that figure among the biggest donors in national elections, as well as the predominance of indirect contributions, are in line with previous political science research analysing corporate campaign contributions in Brazil. This used to be the big picture until 2015: corporate contributions were the main source of funding for electoral campaigns; a few companies and corporate groups donated significant amounts of money which were allocated to a few candidates, who consequently received high sums for their campaigns; political parties had a central role as mediators of corporate contributions, especially those from big donors^(30–32,43–45)^.

The reasons why corporations contribute to elections through political parties and the strategies used to distribute financial resources to political parties and candidates are unknown in this study. It is possible that contributing to the political party could create bonds with the institution, which has more power to develop, shape and introduce public policies. If the choice regarding which candidates were to be financed was made by the political party, resources could have been distributed to those with more chances of being elected. If the choice was made by the corporations, contributions mediated by political parties or other candidates could have been a means to hide a possible relationship with the elected officials.

It is likely that both direct and indirect contributions could create bonds between the corporations and elected officials. Yet, we are not aware of any study addressing whether there are differences in the effect of these types of contribution on the behaviour of elected officials in Brazil. Theoretically, one could say that direct contributions could have a stronger influence on how an elected official votes or defends private interests. However, two hypothetical scenarios must be taken into account. First, if the final destination of the money (candidate) was defined in its origin (corporation), the effect of an indirect contribution in establishing a relationship between the elected official and the corporation could have been the same as that produced by a direct contribution. Second, even if the candidates to be financed were not defined *a priori* by the corporations, both recipients and donors knew who had benefited from the contributions. As a result, although most contributions from sugary drink and sugary drink input industries were made indirectly to candidates, we cannot rule out the possibility that these contributions could indeed buy influence of elected officials on decision-making processes.

The participation from sugary drink industries was greater than that of sugary drink input industries. This result could reflect the considerable economic power of some corporations that produce soft drinks, including the transnational Coca-Cola and Ambev, as well as Cervejaria Petrópolis, which was used as a front company by Construtora Norberto Odebrecht to hide the origin of part of its contributions. In addition, corporations related to agribusiness – in which sugary drink input industries are included – frequently have their owners running up to elections, thus reducing the number of candidates they finance^(34)^. In the case of *Viva Lácteos*, as the association was initiated in 2014, the results of the present analysis could have reflected a period when their associates were not articulated enough for collective action, especially through electoral campaign contributions^(46)^. Also, we cannot rule out that the focus of the CPA from this particular association might be outside the Brazilian Legislature, as by February 2020 no representative had been registered at the 1^st^ Secretariat of the Chamber of Deputies.

The results of the present study also demonstrate that contributions from corporations that are organised in industry associations for collective action – especially those associated with Abir, Unica, and CitrusBR – were more prevalent than those from non-associated ones. This result reflects both the economic power of the associated corporations and their organisation for political action. Attention should be drawn to the fact that industry associations often have a seat in public hearings aimed at discussing public health policies. The most recent one was held in December 2018 to discuss PL 8541/2017 and its attached bills (PL 8675/2017 and PL 10,075/2018), which aimed at increasing taxes on sugary drinks, and in which both Abir and Unica participated. Certainly, the participation of interest groups in electoral processes is part of democratic systems. What is at stake is the imbalance of forces created by the participation of such highly organised special interest groups, which can undermine public interests – including the formulation and implementation of health-related public policies^(39,47)^.

The characteristics of the contributions from sugary drink and sugary drink input industries are aligned with those from the private corporate sector as a whole, as reported in the Brazilian political science literature. They show a preference for male candidates, Caucasians, with a high level of education, who are professional politicians and affiliated to more organised political parties^(32,48,49)^.

Money is a very important resource for electoral success^(32,43,48,50)^. Total revenues of candidates positively correlate with the number of votes, and elected officials usually report having received and spent a significantly higher amount of money in comparison to non-elected candidates. If on the one hand it is true that campaign financing influences the result, on the other expectations related to the chances of victory of the candidates might help increase contributions to their campaigns^(43,48,50–52)^. In an analysis of the 2010 Elections to the Chamber of Deputies, Cervi *et al*.^(48)^ have found that variables such as ‘occupation’, ‘type of political party’ and ‘campaign financing’ mutually reinforce each other. Candidates who referred themselves as being professional politicians, in special those who were seeking re-election, were more likely to be elected. These professional politicians also tend to concentrate on bigger political parties and to get more revenues for their campaigns. Therefore, money is only one among a set of variables that can influence electoral results and which are not always easily measurable.

Despite not being possible to establish a causal relationship between the electoral campaign contributions and the electoral result, those who have received contributions from sugary drink and/or sugary drink input industries are significantly more likely to be elected than their counterparts. As a result, almost half of the elected Federal Deputies have a history of campaign contributions from at least one corporation that is likely to oppose health-related regulation of sugary drinks. This can have several implications for public health policies. The effects of past electoral campaign contributions are likely not to be limited to how elected officials vote. They could also influence intermediate stages of the decision-making process, such as the sponsorship and reporting of bills, the inclusion of a bill in the voting calendar, the call for public hearings, etc. Indeed, there is evidence that past campaign contributions from Coca-cola and Ambev could have influenced three legislators in favour of private interests in the legislative process of PL 8541/2017 and its attached bills (PL 8675/2017 and PL 10,075/2018). They were involved in a chain of events that clearly helped postpone deliberation in the Committee of the Chamber of Deputies where health-related bills are considered^(39)^.

The main strength of this study was the approach used to identify the corporations related to sugary drink and sugary drink input production that contributed financially for this election. The search of a wide array of economic activities and individual checking of the companies have certainly helped increase the accuracy of the estimated participation of this industry sector. Despite this, there are still three limitations for the selection criteria adopted. The first relates to the use of the current economic activity classification. Information on corporations that had been dissolved could not be checked carefully. Also, a few corporations have changed their economic activity completely, possibly indicating they were front corporations used for money laundry or disguising illegal contributions. In such case, we believe these contributions were not likely to have been used to buy future influence on public policies related to the industry sector herein analysed. The second relates to corporations with multiple economic activities. In such cases, only the contributions made under the registries strictly related to the scope of this analysis were accounted. The third relates to corruption scandals which involved two corporations included in the scope of this analysis. Construtora Norberto Odebrecht S.A. has strategically distributed their campaign contributions throughout the several smaller companies that compose the group, including those that grow sugarcane and produce sugar, in order to conform to the financial limit imposed by law and to divert attention from these contributions. And Cervejaria Petrópolis was used as a front company to distribute both legal and illegal financial resources from Construtora Norberto Odebrecht S.A. in exchange for future contracts between the two corporations^(34)^. The effects of these contributions on the future behaviour of elected officials are unknown. This study has also limitations intrinsic to any analysis of electoral campaign contributions in Brazil. First, slush funds cannot be accounted. Second, it is not possible to identify whether regular citizens who contributed to electoral campaigns had any type of relationship with sugary drink or sugary drink input industries. However, this would not impact results significantly, as financial resources from regular citizens represent the smallest share of campaign contributions. Overall, we believe all the limitations of this study could result in an underestimation of the participation of sugary drink and sugary drink input industries, and not the opposite.

In conclusion, this study has shown substantial electoral campaign contributions from the sugary drink and sugary drink input industries to candidates to the Chamber of Deputies in the 55^th^ Congress. As a result, this sector has contributed to the electoral campaigns of almost half of the elected Federal Deputies. We highlight the predominance of revenues mediated by political parties, from sugary drink industries and from corporations organised in some industry associations. This scenario is worrisome because possibly facilitates access to lobby decision-makers and can help buy influence on legislative outcomes. It should be borne in mind that identifying campaign contributions is not sufficient for understanding the influence of corporations on public health policy processes, as lobbying also plays an important role. Moreover, despite the accountability of electoral campaign contributions in Brazil, there is a lack of transparency in decision-making processes, especially because lobbying activities are not regulated in the country and public consultations are not always held. Finally, although corporate electoral campaign contributions are no longer allowed in the country, medium- and long-term effects of previous contributions on the behaviour of elected officials are unknown. Further research must address whether campaign contributions might have influenced decision-making on legislative proposals that could somehow impact sugary drink industry manufacture and commercial practices.
